# Telomere Length and Bipolar Disorder

**DOI:** 10.1038/npp.2017.125

**Published:** 2017-07-26

**Authors:** Timothy R Powell, Danai Dima, Sophia Frangou, Gerome Breen

**Affiliations:** 1 Social, Genetic and Developmental Psychiatry, Institute of Psychiatry, Psychology and Neuroscience, King’s College London, London, UK; 2 National Institute for Health Research Biomedical Research Centre for Mental Health, Institute of Psychiatry, Psychology and Neuroscience at the Maudsley Hospital and King’s College London, London, UK; 3 Department of Psychology, City, University of London, London, UK; 4 Department of Neuroimaging, Institute of Psychiatry, Psychology and Neuroscience, King’s College London, London, UK; 5 Department of Psychiatry, Icahn School of Medicine at Mount Sinai, New York, NY, USA

## Abstract

Variation in telomere length is heritable and is currently considered a promising biomarker of susceptibility for neuropsychiatric disorders, particularly because of its association with memory function and hippocampal morphology. Here, we investigate telomere length in connection to familial risk and disease expression in bipolar disorder (BD). We used quantitative PCRs and a telomere-sequence to single-copy-gene-sequence ratio method to determine telomere length in genomic DNA extracted from buccal smears from 63 patients with BD, 74 first-degree relatives (49 relatives had no lifetime psychopathology and 25 had a non-BD mood disorder), and 80 unrelated healthy individuals. Participants also underwent magnetic resonance imaging to determine hippocampal volumes and cognitive assessment to evaluate episodic memory using the verbal paired associates test. Telomere length was shorter in psychiatrically well relatives (*p*=0.007) compared with unrelated healthy participants. Telomere length was also shorter in relatives (regardless of psychiatric status; *p*<0.01) and patients with BD not on lithium (*p*=0.02) compared with lithium-treated patients with BD. In the entire sample, telomere length was positively associated with left and right hippocampal volume and with delayed recall. This study provides evidence that shortened telomere length is associated with familial risk for BD. Lithium may have neuroprotective properties that require further investigation using prospective designs.

## Introduction

Telomeres are DNA repeat structures (TTAGGG) at the end of each chromosome that undergo shortening during mitosis (Allsop *et al*, 1992; Stewart *et al*, 2012). Telomere shortening has been associated with exposure to cellular stressors (Saretzki and Von Zglinicki, 2002), lifestyle factors (Valdes *et al*, 2005), and social adversity (Epel *et al*, 2004; Gianaros *et al*, 2007; Shalev *et al*, 2013), whereas telomerase, an enzyme that adds DNA sequence repeats (TTAGGG) onto the 3′ telomeric end, may reverse or mitigate this process (Allsop *et al*, 1992; Stewart *et al*, 2012). Cell senescence or cell death is triggered when a critically short telomere length is reached (Stewart *et al*, 2012). Telomere length is therefore considered a promising biomarker of biological aging and susceptibility to disease (Calado and Young, 2009; Heidinger *et al*, 2011).

The relationship between telomere length and psychiatric disorders is a topic of much interest but also uncertainty (Eitan *et al*, 2014). Previous studies have reported shortened peripheral telomere length in major depressive disorder (MDD) (Verhoeven *et al*, 2016; Hartmann *et al*, 2010; Lung *et al*, 2007), schizophrenia (SCZ) (Yu *et al*, 2008), as well as different forms of dementia (Mouiha *et al*, 2011; Rabinovici *et al*, 2007). In bipolar disorder (BD), individual studies have reported both reduced (Barbé-Tuana *et al*, 2016; Lima *et al*, 2015) and increased telomere length in patients compared with unrelated healthy individuals (Martinsson *et al*, 2013; Simon *et al*, 2006). The heterogeneity of the primary studies is reflected in recent meta-analyses that either failed to find an effect of diagnosis (Colpo *et al*, 2015) or reported a small effect (Darrow *et al*, 2016). In post-mortem brain tissue, decreased telomere length has been found in the hippocampus of patients with SCZ, BD, and MDD, suggesting that telomere shortening may be more relevant or pronounced in this brain region (Mamdani *et al*, 2015).

Telomere length is associated with brain morphology, particularly the hippocampus (King *et al*, 2014; Nilsonne *et al*, 2015), a brain region that appears vulnerable to multiple disease mechanisms (Hibar *et al*, 2016; Rabinovici *et al*, 2007; Schmaal *et al*, 2016; van Erp *et al*, 2016; Mouiha *et al*, 2011) that is also capable of neurogenesis (Barnes *et al*, 2009; Spalding *et al*, 2013). Telomere length is also associated with memory function (Valdes *et al*, 2010) including episodic memory that is closely linked to hippocampal function (Dickerson and Eichenbaum, 2010; Kühn and Gallinat, 2014; Van Petten, 2004). Telomere shortening may therefore represent a common biological mechanism linking hippocampal abnormalities and deficits in episodic memory, both of which have been consistently reported in psychiatric disorders (Bora *et al*, 2009, 2013; Bourne *et al*, 2013; Lim *et al*, 2013; Szöke *et al*, 2008).

In the case of BD, psychotropic treatment may influence telomere length. There is convincing evidence that patients on long-term lithium treatment have longer telomeres (Martinsson *et al*, 2013; Squassina *et al*, 2016), whereas the case regarding antidepressants remains equivocal (Verhoeven *et al*, 2017; Bersani *et al*, 2015; Soeiro-de-Souza *et al*, 2014). The relationship between telomere length and BD is therefore complex, implicating both disease- and treatment-related mechanisms. One way to disentangle these effects is to examine first-degree relatives of patients, as telomere length shows high heritability (Slagboom *et al*, 1994). Accordingly, we examined telomere length in remitted patients with BD, first-degree relatives of patients, and unrelated healthy comparison individuals. We further investigated the effect of psychotropic treatment on telomere length and the association between telomere length with hippocampal volume and episodic memory.

## Materials and methods

Buccal DNA was available from 217 individuals of white British ancestry who had participated in the Vulnerability to Bipolar Disorders Study (VIBES), described previously (Delvecchio *et al*, 2015; Dima *et al*, 2013, 2016; Forcada *et al*, 2011; Frangou, 2009, 2011; Jogia *et al*, 2011, 2012; Kempton *et al*, 2009; Lelli-Chiesa *et al*, 2011; Perrier *et al*, 2011; Pompei *et al*, 2011a, 2011b; Ruberto *et al*, 2011; Takahashi *et al*, 2010). The VIBES sample includes (1) patients who fulfill criteria for BD-I based on the Diagnostic and Statistical Manual of Mental Disorders, 4th edition, revised (DSM-IV) (American Psychiatric Association, 1994) (2) first-degree relatives and, (3) unrelated healthy individuals without a personal or family history of psychiatric disorders. Patients and relatives were screened to exclude pedigrees with schizophrenia or schizophrenia spectrum disorders. Exclusion criteria for all participants were current and hereditary neurological disorders, DSM-IV lifetime drug or alcohol dependence or drug or alcohol abuse in the preceding 6 months, and contraindications to magnetic resonance imaging (MRI). Trained psychiatrists and clinical psychologists respectively conducted clinical interviews and cognitive assessments. Diagnostic assessments were based on the Structured Clinical Interview for DSM-IV disorders (First *et al*, 2002a, 2002b) and psychopathology was rated using the Hamilton Depression Rating Scale (HDRS; Hamilton, 1960) and the Young Mania Rating Scale (YMRS; Young *et al*, 1978). The study received institutional ethical approval. All individuals provided written informed consent before participation.

The study sample comprised 63 patients with BD, 74 first-degree relatives (siblings=35; offspring=39), and 80 unrelated healthy volunteers (Table 1 and Supplementary Tables S1 and S2); 27 patients with BD and 8 relatives were unrelated to any other participant in the database. Of the first-degree relatives, 21 had a lifetime diagnosis of MDD and 4 of Anxiety Disorders (Supplementary Table S1). All participants with psychiatric diagnoses were in remission at the time of study enrolment defined as a HDRS and YMRS score below 7 (Table 1), in accordance with the criteria set by the task force of International Society for Bipolar Disorders (Tohen *et al*, 2009). All but 4 patients with BD were medicated as detailed in Table 1. In addition, 15 of the 25 relatives with non-BD psychiatric diagnoses were prescribed antidepressants as monotherapy at the time of study participation (Table 1). Relatives with non-BD diagnoses who were not on treatment had been either medication naive (*n*=7) or had not received any psychotropic treatment for >1 year.

### Cognitive Assessment

In all participants, an estimate of general intellectual ability was obtained using the Wechsler Adult Intelligence Scale-Revised (Wechsler, 1981) and episodic memory was assessed using verbal paired associates (VPA) test from the Wechsler Memory Scale-Third Edition (Wechsler, 1997). This is the most widely used instrument for the assessment of hippocampus-linked memory (Van Petten, 2004). Scaled scores for VPA-immediate and VPA-delayed recall were used in the analyses.

#### Determination of telomere length

Buccal DNA was extracted using a standardized procedure (Freeman *et al*, 2003). DNA samples had good purity ratios (260/280 ratios between 1.7 and 1.9), as measured using the Nanodrop, ND1000 (Thermoscientific, Wilmington, DE). Telomere length was quantified using quantitative real-time PCR (qPCR) assays as previously described (Cawthon, 2009; Vincent *et al*, 2017) performed on the ABI Prism 7900HT Sequence Detection System, with the output generated using SDS Software version 2 (details in Supplementary Material and Supplemental Figure S1). Telomere lengths are reported as relative ratios of the copy number of telomere DNA (TTAGGG) to a single-copy gene (albumin). The telomere length was normally distributed in the entire sample (Kolmogorov–Smirnov *p*=0.09). Eleven specimens were excluded because they either failed quality control (*n*=4) or were identified as outliers (*n*=7) (telomere length >2 SD). The telomere length was negatively associated with age in the entire sample (*β*=−0.18; *t*=−2.72; *p*=0.007) but not with sex (*β*=0.07; *t*=1.09; *p*=0.29). Further analyses showed that the regression slopes were not statistically different by sex or diagnostic group as detailed in Supplementary Material (Supplementary Figure S2 and Supplementary Tables S3–S5). Following linear regression of age and sex, the standardized residuals of the telomere length, referred to as age- and sex-adjusted telomere length, were used in most downstream analyses.

### MR Imaging

High-resolution T1-weighted whole-brain MR images were obtained on a GE Signa HD 1.5T MR imaging system using an inversion recovery prepared, spoiled gradient-echo sequence. Whole-brain coverage was obtained in axial orientation with slice thickness of 1.5 mm, repetition time of 18 ms, echo time of 5.1 ms, flip angle of 20°, field of view=240 × 180 mm, and voxel dimensions=0.9375 × 0.9375 × 1.5 mm. Following preprocessing, we used FreeSurfer, version 5.3.0 (http://surfer.nmr.mgh.harvard.edu/), a widely used and validated software, to segment and respectively quantify total intracranial and left and right hippocampal volume. Segmented regions were visually inspected and statistically evaluated for outliers.

### Statistical Analysis

Group differences were examined using analysis of variance (followed by *post hoc* tests) and independent *t*-tests, as appropriate. Family membership was modeled as a repeated measure. Bivariate associations were assessed using Spearman’s correlation coefficient and regression analyses were used to model the contribution of multiple predictors. We first examined the effect of potential confounders, namely medication in patients and the relatives and the effect of psychiatric status (with regard to non-BD diagnoses) in the relatives to determine the number of groups and variables to be considered. Analyses were performed in SPSS (Version 22, IBM, New York, NY).

## Results

### Examination of Confounders

#### Telomere length and medication in patients with BD

Details on medication are shown in Table 1. In patients with BD, the mean duration of treatment with lithium was 3.8 years (range: 6 months to 40 years) and the mean dose was 904 mg (range 600–1200 mg). Lithium dose and treatment duration did not correlate with telomere length (*p*>0.13). Lithium treatment status was not associated with differences in age of onset, or HDRS and YMRS scores (*p*>0.94). Only four patients with BD who were not on current treatment with lithium had been prescribed this medication at some point in the past.

We then examined the effect of medication (on lithium *vs* not on lithium, on antidepressants *vs* not on antidepressants; typical antipsychotic, atypical antipsychotic, none; carbamazepine, lamotrigine, sodium valproate, none) on age- and sex-adjusted telomere length in patients with BD. We first tested the effect of each individual class and then considered all classes together. Treatment with lithium was associated with longer telomeres (*t*_60_=−2.24, *p*=0.03). We found no effect of antidepressants (*t*_60_=1.13, *p*=0.16), antipsychotics (F_2, 60_=0.57, *p*=0.57), or anticonvulsants (F_3, 60_=1.69, *p*=0.15). When all medications and their interactions were considered, there was still an overall effect of lithium (F_1_=4.01, *p*=0.04), but the main effects and interactions with the other medication classes were not significant (*p*>0.22).

#### Telomere length and medication in relatives of patients with BD

Relatives had only been exposed to antidepressants. A multiple regression analysis did not support an association between telomere length and antidepressant treatment (*β*=−0.02, *t*=−0.33; *p*=0.73) in relatives; in the same model we found a significant association with age (*β*=−0.19, *t*=−2.37; *p*=0.01) but not sex (*β*=0.10, *t*=1.32; *p*=0.18). Moreover, age-and sex-adjusted telomere length did not differ between relatives based on their antidepressant exposure (F_1, 63_=1.79, *p*=0.18). Further analyses on the association between antidepressants and telomere length can be found in Supplementary Material.

#### Telomere length and psychiatric status in relatives of patients with BD

Age- and sex-adjusted telomere length differed between relatives with psychiatric diagnoses, psychiatrically well relatives, and healthy volunteers (F_2, 154_=4.24, *p*=0.01). The *post hoc* Bonferroni corrected pairwise tests showed that healthy relatives had significantly shorter telomere length than healthy volunteers (*p*=0.02), whereas no other pairwise comparison was significant (*p*>0.20).

### Primary Analyses

Having established which confounders were relevant, we proceeded to carry out a series of hypothesis-driven analyses.

#### Telomere length in patients with BD and first-degree relatives

**Figure 1:**
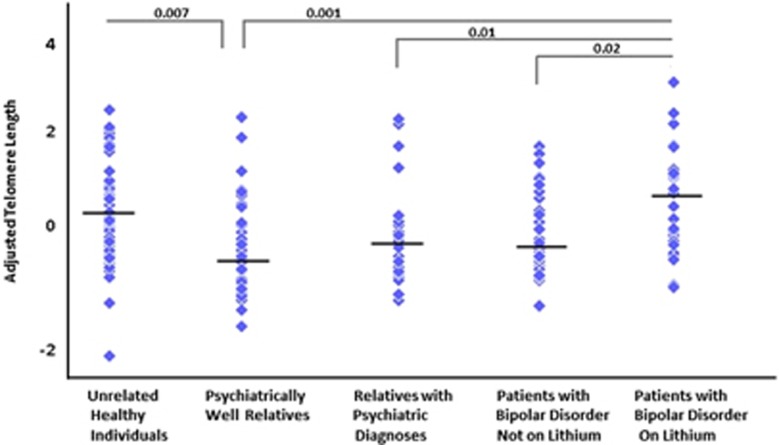
Scatterplot showing the distribution of adjusted telomere length in the study sample. Compared with unrelated healthy individuals, age- and sex-adjusted telomere length was shorter in psychiatrically well relatives and in relatives with psychiatric diagnoses relative to controls (*p*=0.07; not shown in figure). Lithium-treated patients with BD had longer telomere length compared with relatives, regardless of psychiatric status, and patients with BD not on lithium.

Based on the results above we considered five groups in our final analysis, namely, unrelated healthy participants, psychiatrically well relatives, relatives with psychiatric diagnoses, patients with BD on lithium, and patients with BD not on lithium. We found an overall effect of group on age- and sex-adjusted telomere length (F_4, 217_=3.79, *p*=0.005). The *post hoc* Bonferroni corrected pairwise tests showed that compared with unrelated healthy participants, telomere length was shorter in psychiatrically well relatives (*p*=0.007; Figure 1) and relatives with psychiatric diagnoses, although at nominal statistical significance (*p*=0.07). Lithium-treated patients with BD had longer telomere length compared with psychiatrically well relatives (*p*=0.001), relatives with psychiatric diagnoses (*p*=0.01), and patients with BD not on lithium (*p*=0.02); all other pairwise comparisons were not significant.

#### Telomere length and hippocampal volume

The mean and SD of the hippocampal volumes are shown in Table 1 and Supplementary Table S10. There was no effect of group (ie, healthy volunteers, psychiatrically well relatives, relatives with psychiatric diagnoses, patients with BD on lithium, patients with BD not on lithium) on intracranial volume (ICV) (F_2, 162_=0.98, *p*=0.37) and no effect of age (F_1, 162_=0.59, *p*=0.44) but a significant effect of sex (F_1, 162_=4.48, *p*=0.03). There was no significant effect of group on hippocampal volumes (left F_4, 173_=0.81, *p*=0.51; right F_4, 173_=1.59, *p*=0.17); the effects of age and sex were significant (*p*<0.001) but not the group × sex × age interaction (*p*>0.50). We found no significant correlation between hippocampal volumes and lithium dose or duration of lithium treatment (*p*>0.14) in patients with BD; we found no significant correlation between hippocampal volumes and antidepressant treatment in patients with BD or relatives (*p*>0.14).

**Figure 2:**
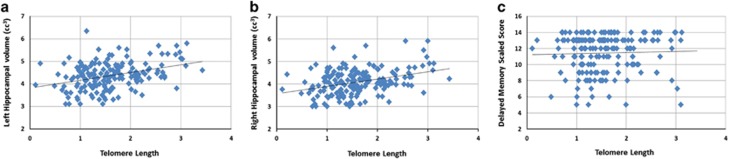
Scatterplot of the association between telomere length, hippocampal volume, and delayed memory in the study sample. In the entire sample, telomere length was positively associated with left (a) and (b) right hippocampal volume and with (c) delayed recall in the verbal paired associates test.

We found no difference in the slopes between telomere length and left and right hippocampal volumes with respect to group (Supplementary Figures S3 and S4 and Supplementary Tables S6 and S7) or sex (Supplementary Figures S5 and S6 and Supplementary Tables S8 and S9). Telomere length explained a substantial amount of the variance of the left (adjusted *R*^2^=0.21, *β*=0.46, *p*<0.001, 95% confidence intervals: 0.32–0.58) and right (adjusted *R*^2^=0.22, *β*=0.47, *p*<0.001, 95% confidence intervals: 0.31–0.56) hippocampal volume (Figure 2).

#### Telomere length and episodic memory

The mean and SD of the memory variables are shown in Table 1. There was no significant main effect of group (healthy volunteers, psychiatrically well relatives, relatives with psychiatric diagnoses, patients with BD on lithium, patients with BD not on lithium) on IQ (F_4, 184_=2.36, *p*=0.20). There was a significant effect of group on VPA-immediate recall (F_4, 184_=2.59, *p*=0.04). Nonlithium-treated patients with BD (*p*=0.008) and relatives with psychiatric diagnoses performed worse than healthy volunteers (*p*=0.05). Similarly, there was a significant main effect of group on VPA-delayed recall (F_4, 184_=8.51, *p*<0.001). Compared with unrelated healthy volunteers, delayed recall was reduced in psychiatrically well relatives (*p*<0.001), relatives with psychiatric diagnoses (*p*<0.001), and nonlithium-treated patients with BD (*p*=0.05).

We found no difference in the slopes between telomere length and VPA-immediate and VPA-delayed recall with respect to group (Supplementary Figures S7 and S9 and Supplementary Tables S11 and S13) or sex (Supplementary Figures S8 and S10 and Supplementary Tables S12 and S14). Telomere length explained a nonsignificant amount of the variance in VPA-immediate recall (adjusted *R*^2^=0.004; *β*=0.09; *p*=0.19, 95% confidence intervals: −0.26, 1.31). Telomere explained a small but significant amount of the variance for VPA-delayed recall (adjusted *R*^2^=0.02 0; *β*=0.14, *p*=0.05; 95% confidence intervals: −0.003, 1.25) (Figure 2).

#### Telomere length and clinical features

We examined correlations between age- and sex-adjusted telomere length and severity of manic and depressive psychopathology, number of episodes (total, manic, depressive, mixed), and age of onset. None was significant (*ρ*<0.15, *p*>0.10).

## Discussion

This is the first study to date to demonstrate a link between shorter telomere length and familial risk for BD. Lithium treatment was associated with telomere length such that patients on long-term lithium treatment had longer telomeres compared with relatives and patients with BD who were not treated with lithium. In the entire sample, telomere length was also associated with larger hippocampal volume and better episodic memory.

Our results suggest that shorter telomere length may be a common factor linking genetic liability to BD to multisystem disorder vulnerability. Shorter telomeres have been associated with multiple adverse health outcomes, primarily cardiovascular disease (Brouilette *et al*, 2003; D’Mello *et al*, 2015; Fitzpatrick *et al*, 2007; O’Donnell *et al*, 2008), type 2 diabetes (O’Donnell *et al*, 2008), age-related cognitive dysfunction (Yaffe *et al*, 2011), and dementia (Panossian *et al*, 2003; Thomas *et al*, 2008). Comorbidities such as hypertension, elevated lipids, poor glycemic control, and diabetes type 2 are more prevalent in patients with BD compared with the general population (Beyer *et al*, 2005; Czepielewski *et al*, 2013; Fiedorowicz *et al*, 2010; Forty *et al*, 2014; McIntyre *et al*, 2006, 2010; Smith *et al*, 2013). Although psychotropic medication may contribute to physical morbidity, cardiometabolic disturbances in patients with BD have been observed independent of medication exposure (Maina *et al*, 2008; Regenold *et al*, 2002). Medical morbidity in relatives of patients with BD is understudied, but according to a recent study, 26% of first-degree relatives of patients with BD self-reported a cardiometabolic disorder as compared with 13% of individuals without a family history of psychiatric disorders (Mothi *et al*, 2015). Patients with BD are also at greater risk of developing dementia later in life compared with patients with nonpsychiatric disorders (da Silva *et al*, 2013; Kessing and Nilsson, 2003) but this association has not been examined in first-degree relatives.

Telomere length was shorter in first-degree relatives compared with unrelated healthy comparison individuals, potentially reflecting the cumulative lifetime burdens of genetic and environmental exposures. Although shorter telomere length has been linked to insufficient telomerase activity (Blackburn, 1991; Lu *et al*, 2013), large population-based studies suggest individuals with shorter telomeres (Epel *et al*, 2008; Farzaneh-Far *et al*, 2010), including those individual experiencing significant distress (Damjanovic *et al*, 2007), may have upregulated telomerase that maintains telomere length. Telomerase activity has not been assessed in individuals with familial risk for BD, but in patients longer telomere length has been associated with lithium-induced increase in telomerase activity (Martinsson *et al*, 2013; Wei *et al*, 2015; Squassina *et al*, 2016). Alternatively, longer telomere length in BD may be predictive of good lithium response and hence long-term lithium treatment. The cross-sectional nature of the current study does not allow us to resolve the direction of causality. We did not find evidence that antidepressants influence telomere length in patients with BD or their relatives, consistent with the lack of such an association reported in larger studies (Verhoeven *et al*, 2017).

We confirmed previously reported associations between telomere length, hippocampal volume (King *et al*, 2014; Nilsonne *et al*, 2015), and episodic memory (Valdes *et al*, 2005) that support the notion that telomere length is a marker of hippocampal vulnerability linked to reduced cell proliferation potential (Wolkowitz *et al*, 2015). Reduction in proliferative potential is likely to affect primarily cells capable of division in the adult brain; these would include neural stem cells located in the dentate gyrus (Gage *et al*, 1995; Palmer *et al*, 1997) and other cells that support neuronal function (eg, microglia, astrocytes, oligodendrocytes, and pericytes). In preclinical studies, telomerase induction has been shown to reverse tissue degeneration (Jaskelioff *et al*, 2011; Shingu *et al*, 2015) and restore proliferative potential of neuronal progenitor cells (Jaskelioff *et al*, 2011) that may account for the association between lithium treatment and decreased risk of developing dementia in patients with BD (da Silva *et al*, 2013; Kessing *et al*, 2008).

There are several methodological considerations pertinent to this study. Telomere length was ascertained from buccal rather than brain tissue. However, previous studies have found that this is an acceptable surrogate as genetic influences on the regulation of telomere length appear tissue independent (Dlouha *et al*, 2014; Friedrich *et al*, 2000). We conducted a number of analyses to estimate and subsequently model the contribution of potential confounding (eg, medication) and moderating variables (eg, age). No correction for multiple comparisons was applied to these analyses as the intention was to identify all potential sources of variance in this data set and account for them in hypothesis testing. The results of our hypothesis-driven analyses survive Bonferroni correction. Medication adherence was based on participants’ self-report. We considered these reports largely valid given that patients had remained on their prescribed medication for long periods and were in remission at study entry. As telomere length changes over time, longitudinal designs are critical in delineating the trajectories of change. However, general population samples have found no evidence of accelerated telomere shortening over follow-up periods of 5–10 years in people with depression or anxiety disorders (Hoen *et al*, 2011; Verhoeven *et al*, 2016, 2017). Telomere shortening has been associated with adversity (Epel *et al*, 2004; Gianaros *et al*, 2007; Shalev *et al*, 2013), oxidative stress and inflammation (Masi *et al*, 2012; O'Donovan *et al*, 2011; Saretzki and Von Zglinicki, 2002), insulin resistance and type 2 diabetes (Demissie *et al*, 2006), obesity, and smoking (Valdes *et al*, 2005). In addition, telomere length may also be influenced by other genetic risk factors for BD. Examination of the complex interface between these factors, telomere length, and BD in longitudinal studies will further enrich our understanding of the biological mechanisms involved.

To our knowledge, this study provides the first evidence linking telomere length to familial propensity to BD. An increased understanding of telomere biology may lead to potential therapeutic interventions to maintain telomere length or reverse telomere attrition. In clinical practice, it would be advisable to target modifiable risk factors such as smoking, obesity, and stress and promote protective factors relating to healthy lifestyle and physical activity (Cherkas *et al*, 2008). Furthermore, targeting telomerase or other associated proteins may provide novel pharmacological targets that could address both mental and physical morbidity in those predisposed to or suffering from BD.

## Funding and disclosure

The authors declare no conflict of interest.

## Figures and Tables

**Table 1:** Sample Characteristics

	**Patients with BD on lithium** ***N*=28**	**Patients with BD not on lithium**, ***N*=35**	**Relatives with psychiatric diagnoses**, ***N*=25**	**Psychiatrically well relatives** ***N*=49**	**Unrelated healthy individuals**, ***N*=80**
Age (years)^a^	45.53 (10.23)	42.91 (10.65)	30.40 (9.84)	36.59 (14.74)	39.71 (14.82)
Sex, *n* (% male)	13 (46.40)	17 (48.60)	9 (36.0)	23 (46.9)	36 (45)
IQ	116.30 (14.21)	121.19 (21.48)	108.80 (14.25)	118.78 (16.87)	121.82 (19.88)
WMS-VPA: immediate recall	11.86 (1.95)	10.45 (32.26)	9.23 (2.85)	10.97 (3.36)	11.92 (3.16)
WMS-VPA: delayed recall	12.07 (1.24)	10.33 (2.83)	8.71 (4.44)	10.87 (2.85)	12.18 (1.85)
Hamilton Depression Rating Scale^b^	3.53 (4.37)	4.31 (4.87)	1.44 (2.23)	0.23 (0.67)	0.17 (0.61)
Young Mania Rating Scale^b^	1.17 (2.55)	1.17 (2.10)	0.32 (1.14)	0.04 (0.29)	0.15 (0.45)
Age of onset of bipolar disorder (years)	24.36 (7.33)	26.54 (9.27)	NA	NA	NA
Any medication, *n* (%)^c^	28 (100%)	30 (85.70)	15 (20.27)	0	NA
Any antidepressant (*n*)^d^	15 (53.60)	16 (45.70)	15 (20.27)	0	NA
Any antipsychotic (*n*)^e^	11 (39.60)	13 (37.10)	0	0	NA
Any anticonvulsant (*n*)^f^	5 (17.90)	21 (60.00)	0	0	NA
Hippocampal volume, left (cm^3^)	4.41 (0.55)	4.39 (0.49)	4.32 (0.61)	4.33 (0.6)	4.44 (0.60)
Hippocampal volume, right (cm^3^)	4.11 (0.49)	4.07 (0.42)	4.02 (0.55)	3.98 (0.58)	4.41 (0.60)

Abbreviations: BD, bipolar disorder; IQ, intelligence quotient; VPA, verbal paired associates; WMS, Wechsler Memory Scale-III.

All continuous variables are shown as mean (SD); IQ was derived from the Wechsler Adult Intelligence Scale-Revised; Scaled scores reported for both WMS-VPA measures.

a Relatives <patients and controls; *p*<0.03.

b Patients >relatives, controls, all *p*<0.0001.

c 59 Patients were prescribed more than one psychotropic.

d All antidepressants prescribed were serotonin reuptake inhibitors.

e All but three antipsychotics prescribed were second-generation agents.

f Sodium valproate=14; carbamazepine=5; lamotrigine=2; combinations=5.
