# Telomere Length and Bipolar Disorder

**DOI:** 10.1038/npp.2017.239

**Published:** 2017-12-12

**Authors:** Timothy R Powell, Danai Dima, Sophia Frangou, Gerome Breen

**Correction to:** *Neuropsychopharmacology* advance online publication, 26 July 2017; doi:10.1038/npp.2017.125

This article was originally published under NPG’s License to Publish, but has now been made available under a CC BY 4.0 license. The PDF and HTML versions of the paper have been modified accordingly.

