# Supplementary material for: Telomere Length and Bipolar Disorder
Source: Neuropsychopharmacology. 2017 Jul 26;43(2):445–53. doi: 10.1038/npp.2017.125 (PMC5729555; doi:10.1038/npp.2017.125)
Supplement: Supplementary Material [file npp2017125x1.docx]

**Telomere length and bipolar disorder**

1. **Supplemental Methods**

| **Table S1: Sample Characteristics** | | | |
| --- | --- | --- | --- |
|  | **Patients**  **N=63** | **Relatives**  **N=74** | **Unrelated**  **Participants**  **N=80** |
| **Age (years)^a^** | 44.04 (10.33) | 34.50 (12.85) | 39.71 (14.82) |
| **Sex, n (% male)** | 30 (47.61) | 33 (44.59) | 36 (45) |
| **IQ** | 118.80 (18.24) | 115.31 (16.71) | 121.82 (19.88) |
| **WMS-VPA: immediate recall** | 9.92 (2.94) | 10.98 (3.51) | 11.92 (3.16) |
| **WMS-VPA: delayed recall** | 9.78 (2.70) | 10.80 (2.89) | 11.76 (2.25) |
| **Hamilton Depression Rating Scale^b^** | 3.88 (4.67) | 0.66 (1.53) | 0.17 (0.61) |
| **Young Mania Rating Scale^b^** | 1.17 (2.29) | 0.14 (0.72) | 0.15 (0.45) |
| **Age of onset of Bipolar Disorder (years)** | 25.50 (8.38) | n/a | n/a |
| **Any Medication, n (%)^c^** | 59 (93.65) | 15 (20.27) | n/a |
| **Lithium (n)** | 28 (44.44) | 0 | n/a |
| **Any Antidepressant (n)^d^** | 31 (49.20) | 15 (20.27) | n/a |
| **Any Antipsychotic (n)^e^** | 24 (38.09) | 0 | n/a |
| **Any Anticonvulsant (n)^f^** | 26 (41.26) | 0 | n/a |
| **Hippocampal volume, Left (cm^3^)** | 4.39 (0.52) | 4.36 (0.62) | 4.44 (0.60) |
| **Hippocampal volume, Right (cm^3^)** | 4.08 (0.46) | 4.01 (0.47) | 4.41 (0.60) |
| All continuous variables are shown as mean (standard deviation); Intelligence Quotient (IQ) was derived from the Wechsler Adult Intelligence Scale-Revised; WMS= Wechsler Memory Scale-III; VPA=Verbal Paired Associates; Scaled scores reported for both WMS-VPA measures  ^a^ Relatives <Patients and Controls; p<0.03; ^b^ Patients> Relatives, Controls, all p<0.0001; ^c^ 59 patients were prescribed more than one psychotropic; ^d^ all antidepressants prescribed were serotonin reuptake inhibitors; ^e^ all but 3 antipsychotics prescribed were second generation agents; ^f^ sodium valproate=14; carbamazepine=5; lamotrigine=2; combinations=5 | | | |

| **Table S2: Characteristics of Relatives group** | | | |
| --- | --- | --- | --- |
|  | **Relatives**  **N=74** | **Siblings**  **N=35** | **Offspring**  **N=39** |
| **Age (years)^a^** | 34.36 (12.93) | 44.25 (11.48) | 25.30 (5.64) |
| **Sex, n (% male)** | 33 (44.59) | 18 (51.42) | 16 (41.02) |
| **IQ** | 115.31 (16.71) | 114.28 (15.57) | 107.76 (13.39) |
| **Brief Psychiatric Rating Scale^b^** | 24.69 (1.66) | 24.14 (0.42) | 25.20 (2.12) |
| **Hamilton Depression Rating Scale^b^** | 0.63 (1.51) | 0.11 (0.40) | 1.12 (1.92) |
| **Young Mania Rating Scale** | 0.13 (0.71) | 0.5 (0.33) | 0.20 (0.92) |
| **No lifetime psychiatric diagnosis,** **n (%) ^c^** | 49 (67.56) | 29 (82.85) | 20 (51.28) |
| **Major Depressive Disorder,**  **n (%)** | 21 (28.37) | 6 (17.14) | 15 (38.46) |
| **Anxiety Disorder,**  **n (%)** | 4 (5.40) | 0 | 4 (10.25) |
| All continuous variables are shown as mean (standard deviation); Intelligence Quotient (IQ) was derived from the Wechsler Adult Intelligence Scale-Revised; ^a^ Offspring <Siblings,p<0.0001; ^b^ Offspring>Siblings, all p<0.004; ^c^ Offspring>Siblings, p=0.02 | | | |

**Determination of Telomere Length**

Buccal DNA was extracted using a standardised procedure described previously (Freeman et al., 1999). DNA samples had good purity ratios (260/280 ratios of between 1.7–1.9), as measured using the Nanodrop, ND1000 (Thermoscientific, Wilmington, DE). Telomere length was quantified using the output from two separate quantitative real-time polymerase chain reactions (qPCRs) as previously described (Cawthorn, 2009) performed on the ABI Prism 7900HT Sequence Detection System, with the output generated using SDS Software version 2 (details in supplemental material).

The first qPCR assays the telomere repeat region (TTAGGG), and the second qPCR assays a single copy gene (albumin). Reactions were performed in 384-well plates and matched sample/well positions were used across the two reactions. All samples under investigation were tested using three technical replicates, and six no template controls were included on each plate. An eight point genomic DNA (human leukocyte) dilution series (0.47 ng, 0.94 ng, 1.88 ng, 3.75 ng, 7.5 ng, 15 ng, 30 ng, 60 ng) was included in every plate to create a standard curve, which was used to perform absolute quantification of each DNA sample under investigation. Five calibrator samples, containing leukocyte DNA from five separate human subjects, were run in triplicate on each plate in order to correct for inter-plate variability. Both the DNA for the standard curve (highest standard) and the calibrators were prepared in one single batch, aliquoted, and frozen at -20°C, in order to reduce variability. For each run, we thawed a set of calibrator aliquots, and an aliquot of the highest concentration of the standards; with the dilution series prepared fresh on the day.

The qPCR protocol we used is a modified version of a reaction described previously (Cawthorn, 2009). Per reaction, the telomere qPCR mix consisted of: 10.5 uL Precision PLUS SYBR green Mastermix (Primer Design, Southampton, UK), 4.5 uL RNase free water, 12 ng DNA, 1000 nM telg, 5’-ACACTAAGGTTTGGGTTTGGGTTTGGGTTTGGGTTAGTGT-3’ and 800 nM telc, 5’-TGTTAGGTATCCCTATCCCTATCCCTATCCCTATCCCTAACA-3’. The telomere thermocycling conditions consisted of four stages: Step 1: 15 minutes at 95°C; Step 2: 2 cycles at 94°C for 15 seconds and 49°C for 15 seconds; Step 3: 25 cycles at 94°C for 15 seconds, 62°C for 10 seconds and 73°C for 15 seconds (data collection); Step 4: dissociation curve (primer specificity detection).

The albumin qPCR mix consisted of the same ingredients as above, but using primers for the albumin gene. We used 765 nM of the forward primer (albu): 5’-CGGCGGCGGGCGGCGCGGGCTGGGCGGAAATGCTGCACAGAATCCTTG-3’ and 900 nM of the reverse primer (albd): 5’-GCCCGGCCCGCCGCGCCCGTCCCGCCGGAAAAGCATGGTCGCCTGTT-3’. The albumin thermocycling conditions consisted of four stages: Step1: 15 minutes at 95°C; Step 2: 2 cycles at 94°C for 15 seconds and 49°C for 15 seconds; Step 3: 33 cycles at 94°C for 15 seconds, 62°C for 10 seconds and 88°C for 15 seconds (data collection); Step 4: dissociation curve (primer specificity detection).

For a sample to be included in downstream data analysis, at least two of the cycle threshold (Ct) technical triplicates needed to achieve a standard deviation of less than 0.5. Remaining Ct values were then related to absolute quantities as part of a standard curve, creating Cq values. The mean Cq value for remaining technical replicates was calculated for each sample and was divided by the mean Cq of the five calibrator samples to create “mean adjusted Cq values” (adjusted for inter-plate variability). Relative telomere length (RTL) was then calculated by dividing the mean adjusted Cq value for each sample in the telomere reaction by the mean adjusted Cq value for each sample in the albumin reaction.

**Telomere Quality Control Checks**

All standard curves showed an R2 of greater than 0.985 between known DNA quantity and Ct values. None of the no-template controls showed amplification in any of our plates. Dissociation curves (melting curves) revealed a clear single peak, confirming amplification specificity across all of our plates, Figure S1. The mean PCR efficiency for the telomere reaction was 100%, and the mean PCR efficiency for the albumin reaction was 95%. The mean adjusted Cq values for the positive controls were used to estimate inter-plate variability by generating coefficient of variation (CV) estimates. Results revealed acceptably low mean CV’s for the telomere repeat region (CV = 3.6%), the albumin single copy gene (CV = 6.4%), and the telomere/albumin ratio (CV = 5.8%). 11 samples (7% of total sample) were excluded from downstream analysis because they either failed quality control or were identified as outliers.


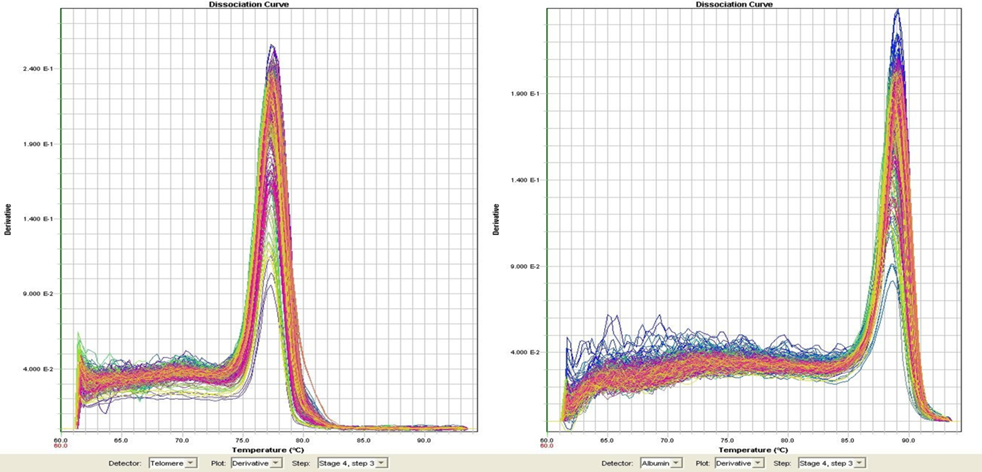


**Figure S1**: Dissociation curves produced during our qPCR reactions revealed specific amplification of the telomere repeat region (left) and albumin gene (right); output from the ABI Prism SDS Software version 2.2.

1. **Supplemental Analyses**

| **Table S3: Regression analyses of telomere length and sex per group** | | | | | | | | |
| --- | --- | --- | --- | --- | --- | --- | --- | --- |
| **Group** | **Overall Model** | | **Standardized**  **Coefficient** | | | **Bootstrap Coefficient and 95% confidence intervals (CI)** | | |
|  | **Adjusted**  **R^2^** | **p-value** | **Beta** | **t-value** | **p-value** | **Beta** | **Lower CI** | **Higher CI** |
| **Unrelated healthy participants** | 0.05 | 0.24 | 0.13 | 1.18 | 0.24 | 0.14 | -0.7 | 0.34 |
| **Relatives with psychiatric diagnoses** | 0.08 | 0.29 | 0.19 | 1.31 | 0.25 | 0.15 | -0.3 | 0.45 |
| **Psychiatrically Well**  **Relatives** | 0.01 | 0.22 | 0.17 | 1.21 | 0.22 | 0.16 | -0.9 | 0.41 |
| **Patients BD not on Li** | 0.02 | 0.21 | 0.16 | 1.28 | 0.21 | 0.14 | -0.1 | 0.55 |
| **Patients BD on Li** | 0.02 | 0.22 | 0.14 | 1.21 | 0.22 | 0.12 | -0.9 | 0.31 |
|  |  |  |  |  |  |  |  |  |

| **Table S4: Regression analyses of telomere length and age per group** | | | | | | | | |
| --- | --- | --- | --- | --- | --- | --- | --- | --- |
| **Group** | **Overall Model** | | **Standardized**  **Coefficient** | | | **Bootstrap Coefficient and 95% confidence intervals (CI)** | | |
|  | **Adjusted**  **R^2^** | **p-value** | **Beta** | **t-value** | **p-value** | **Beta** | **Lower CI** | **Higher CI** |
| **Unrelated healthy participants** | 0.03 | 0.05 | -0.21 | -1.95 | 0.05 | -0.007 | -0.01 | 0.001 |
| **Relatives with psychiatric diagnoses** | -0.03 | 0.54 | -0.11 | -0.53 | 0.55 | -0.006 | -0.03 | 0.02 |
| **Psychiatrically Well**  **Relatives** | 0.09 | 0.01 | -0.33 | -2.44 | 0.01 | -0.01 | -0.02 | -0.004 |
| **Patients BD not on Li** | -0.02 | 0.76 | -0.05 | -0.29 | 0.76 | -0.002 | -0.01 | 0.01 |
| **Patients BD on Li** | 0.10 | 0.05 | -0.36 | -2.02 | 0.05 | -0.02 | -0.05 | 0.002 |

|  |
| --- |
|  |

**Figure S2. Comparison of regression slopes for age and telomere length;** the data supported a linear model; details also in Table S5. Group 0=unrelated healthy controls; Group 1=relatives with psychiatric disorders; Group 2=psychiatrically well relatives; Group 3=patients with bipolar disorder not on lithium; Group 4=patients with bipolar disorder on lithium; Age in years****

| **Table S5 Comparisons of regression slopes for age and telomere length by group: Model Summary** | | | | | |
| --- | --- | --- | --- | --- | --- |
| **Source** | **Sum of Squares** | **Degrees of Freedom** | **Mean Square** | **F-Ratio** | **P-Value** |
| **age** | 2.51 | 1 | 2.51 | 7.58 | 0.006 |
| **Intercepts** | 5.06 | 4 | 1.26 | 3.83 | 0.005 |
| **Slopes** | 1.18 | 4 | 0.29 | 0.90 | 0.46 |
| **Model** | 8.76 | 9 |  |  |  |

**Figure S3.** **Comparison of regression slopes for the left hippocampal volume and telomere length by group**; details also in Table S6. Group 0=unrelated healthy controls; Group 1=relatives with psychiatric disorders; Group 2=psychiatrically well relatives; Group 3=patients with bipolar disorder not on lithium; Group 4=patients with bipolar disorder on lithium; volume in cc^3^

| **Table S6 Comparisons of regression slopes for telomere length and left hippocampal volume by group: Model Summary** | | | | | |
| --- | --- | --- | --- | --- | --- |
| **Source** | **Sum of Squares** | **Degrees of Freedom** | **Mean Square** | **F-Ratio** | **P-Value** |
| **Telomere length** | 13.60 | 1 | 13.60 | 47.13 | 0.0000 |
| **Intercepts** | 0.14 | 4 | 0.03 | 0.12 | 0.97 |
| **Slopes** | 0.79 | 4 | 0.19 | 0.69 | 0.60 |
| **Model** | 14.53 | 9 |  |  |  |

**Figure S4**. **Comparison of regression slopes for the right hippocampal volume and telomere length by group**; details also in Table S7. Group 0=unrelated healthy controls; Group 1=relatives with psychiatric disorders; Group 2=psychiatrically well relatives; Group 3=patients with bipolar disorder not on lithium; Group 4=patients with bipolar disorder on lithium; volume in cc^3^

| **Table S7 Comparisons of regression slopes for telomere length and right hippocampal volume by group: Model Summary** | | | | | |
| --- | --- | --- | --- | --- | --- |
| **Source** | **Sum of Squares** | **Degrees of Freedom** | **Mean Square** | **F-Ratio** | **P-Value** |
| **Telomere length** | 12.71 | 1 | 12.71 | 49.86 | 0.0000 |
| **Intercepts** | 0.39 | 4 | 0.09 | 0.39 | 0.81 |
| **Slopes** | 0.84 | 4 | 0.21 | 0.83 | 0.50 |
| **Model** | 13.96 | 9 |  |  |  |

**Figure S5.** **Comparison of regression slopes for the left hippocampal volume and telomere length by sex**; details also in Table S8. Male=1; Female=2; volume in cc^3^

| **Table S8 Comparisons of regression slopes for left hippocampal volume and telomere length by sex: Model Summary** | | | | | |
| --- | --- | --- | --- | --- | --- |
| **Source** | **Sum of Squares** | **Degrees of Freedom** | **Mean Square** | **F-Ratio** | **P-Value** |
| **Telomere length** | 13.60 | 1 | 13.60 | 52.93 | 0.0000 |
| **Intercepts** | 3.918 | 1 | 3.91 | 15.25 | 0.0001 |
| **Slopes** | 0.62 | 1 | 0.62 | 2.44 | 0.12 |
| **Model** | 18.14 | 3 |  |  |  |

**Figure S6**. **Comparison of regression slopes for the right hippocampal volume and telomere length by sex**; details also in Table S9. Male=1; Female=2; volume in cc^3^

| **Table S9 Comparisons of regression slopes for left hippocampal volume and telomere length by sex: Model Summary** | | | | | |
| --- | --- | --- | --- | --- | --- |
| **Source** | **Sum of Squares** | **Degrees of Freedom** | **Mean Square** | **F-Ratio** | **P-Value** |
| **Telomere length** | 12.71 | 1 | 12.71 | 53.63 | 0.0000 |
| **Intercepts** | 2.70 | 1 | 2.70 | 11.42 | 0.0009 |
| **Slopes** | 0.03 | 1 | 0.03 | 0.15 | 0.69 |
| **Model** | 15.46 | 3 |  |  |  |

| **Table S10. Hippocampal volume and episodic memory in relatives of patients depending on psychopathology and antidepressant treatment and unrelated healthy participants** | | | | | |
| --- | --- | --- | --- | --- | --- |
|  | **Psychiatrically Well Relatives**  **N=41** | **Relatives with Psychiatric Diagnoses**  **N=23** | **Medicated Relatives**  **with Psychiatric Diagnoses**  **N=15** | **Unmedicated**  **Relatives with Psychiatric Diagnoses**  **N=10** | **Unrelated Participants**  **N=73** |
| **Hippocampal volume,**  **Left (cm^3^)** | 4.23 (0.63) | 4.32 (0.61) | 4.20 (0.70) | 4.47 (0.54) | 4.44 (0.60) |
| **Hippocampal volume,**  **Right (cm3)** | 3.92 (0.58) | 4.02 (0.55) | 3.95 (0.66) | 4.12 (0.48) | 4.41 (0.60) |
| **WMS-VPA: immediate recall** | 10.90 (3.01) | 9.23 (2.86) | 9.89 (3.44) | 8.60 (1.11) | 11.92 (3.16) |
| **WMS-VPA: delayed recall** | 10.39 (3.1) | 8.71 (4.44) | 8.80 (4.44) | 8.00 (4.58) | 11.76 (2.25) |
| Variables shown as mean (standard deviation); WMS= Wechsler Memory Scale-III; VPA=Verbal Paired Associates; Scaled scores reported for both WMS-VPA measures; medicated relatives were on antidepressant monotherapy | | | | | |

**
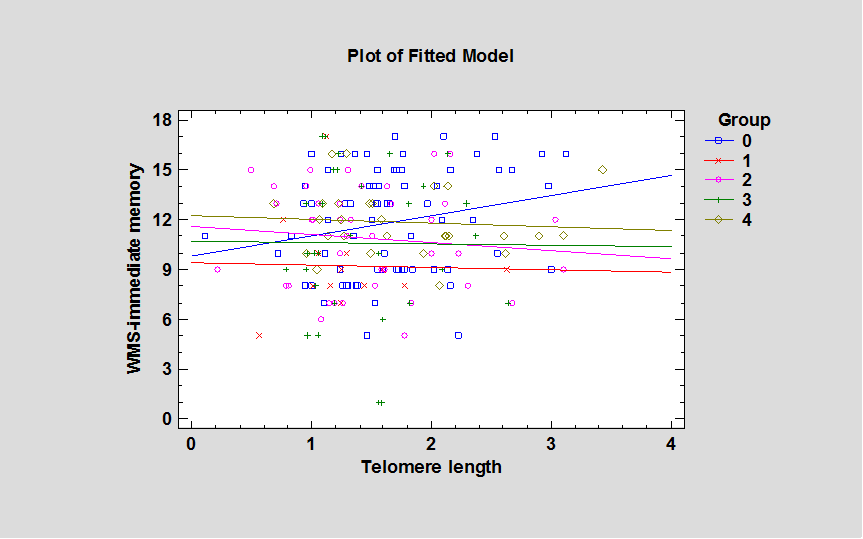
Figure S7**. **Comparison of regression slopes for the VPA-immediate recall and telomere length by group**; details also in Table S11. Group 0=unrelated healthy controls; Group 1=relatives with psychiatric disorders; Group 2=psychiatrically well relatives; Group 3=patients with bipolar disorder not on lithium; Group 4=patients with bipolar disorder on lithium; WMS=Wechsler Memory Scale; VPA=Verbal paired associates

| **Table S11 Comparisons of regression slopes VPA-immediate memory and telomere length by group: Model Summary** | | | | | |
| --- | --- | --- | --- | --- | --- |
| **Source** | **Sum of Squares** | **Degrees of Freedom** | **Mean Square** | **F-Ratio** | **P-Value** |
| **VPA-immediate memory** | 17.78 | 1 | 17.78 | 1.71 | 0.19 |
| **Intercepts** | 93.54 | 4 | 23.38 | 2.25 | 0.06 |
| **Slopes** | 35.22 | 4 | 8.80 | 0.85 | 0.49 |
| **Model** | 146.54 | 9 |  |  |  |

**Figure S8. Comparison of regression slopes for the VPA-immediate recall and telomere length by sex**; details also in Table S12. Group 1=Male, Group 2=Female; WMS=Wechsler Memory Scale; VPA=Verbal paired associates

| **Table S12 Comparisons of regression slopes VPA-immediate memory and telomere length by sex: Model Summary** | | | | | |
| --- | --- | --- | --- | --- | --- |
| **Source** | **Sum of Squares** | **Degrees of Freedom** | **Mean Square** | **F-Ratio** | **P-Value** |
| **VPA-immediate memory** | 17.78 | 1 | 17.78 | 1.66 | 0.19 |
| **Intercepts** | 6.07 | 1 | 6.07 | 0.57 | 0.45 |
| **Slopes** | 5.82 | 1 | 5.82 | 0.54 | 0.46 |
| **Model** | 29.68 | 3 |  |  |  |

**Figure S9. Comparison of regression slopes for the VPA-delayed recall and telomere length by group**; details also in Table S13. Group 0=unrelated healthy controls; Group 1=relatives with psychiatric disorders; Group 2=psychiatrically well relatives; Group 3=patients with bipolar disorder not on lithium; Group 4=patients with bipolar disorder on lithium; WMS=Wechsler Memory Scale; VPA=Verbal paired associates

| **Table S13 Comparisons of regression slopes VPA-delayed memory and telomere length by group: Model Summary** | | | | | |
| --- | --- | --- | --- | --- | --- |
| **Source** | **Sum of Squares** | **Degrees of Freedom** | **Mean Square** | **F-Ratio** | **P-Value** |
| **VPA-delayed memory** | 25.42 | 1 | 25.42 | 4.30 | 0.03 |
| **Intercepts** | 179.6 | 4 | 44.90 | 7.59 | 0.0000 |
| **Slopes** | 11.18 | 4 | 2.79 | 0.47 | 0.75 |
| **Model** | 216.22 | 9 |  |  |  |

**Figure S10. Comparison of regression slopes for the VPA-delayed recall and telomere length by sex**; details also in Table S14. Group 1=Male, Group 2=Female; WMS=Wechsler Memory Scale; VPA=Verbal paired associates

| **Table S14 Comparisons of regression slopes VPA-immediate memory and telomere length by sex: Model Summary** | | | | | |
| --- | --- | --- | --- | --- | --- |
| **Source** | **Sum of Squares** | **Degrees of Freedom** | **Mean Square** | **F-Ratio** | **P-Value** |
| **VPA-immediate memory** | 25.42 | 1 | 25.42 | 3.76 | 0.05 |
| **Intercepts** | 1.56 | 1 | 1.56 | 0.23 | 0.63 |
| **Slopes** | 2.14 | 1 | 2.14 | 0.32 | 0.57 |
| **Model** | 29.14 | 3 |  |  |  |

**Effect of antidepressant treatment**

Thirty-one patients with BD (n=31) were on antidepressants and of these 15 were also on lithium. After excluding patients on lithium, we analysed the data with age-and-sex adjusted telomere length as the dependent variable, treatment with antidepressant (on antidepressants vs not on antidepressant) and diagnostic group (patients with BD, relatives and unrelated healthy participants) as independent factors. We found no effect of antidepressant treatment (F_1_=2.12; p=0.14) or antidepressant treatment by group interaction (F_1_=0.02; p=0.88).

**References**

Cawthon RM (2009). Telomere length measurement by a novel monochrome multiplex quantitative PCR method. Nucleic Acids Res 37, e21: doi: 10.1093/nar/gkn1027
